# Oxidative Stress-Induced Silver Nano-Carriers for Chemotherapy

**DOI:** 10.3390/ph15121449

**Published:** 2022-11-22

**Authors:** Minh Phuong Nguyen, Duy Phong Pham, Dukjoon Kim

**Affiliations:** 1 School of Chemical Engineering, Sungkyunkwan University, Suwon 16419, Republic of Korea; 2 Department of Electrical and Computer Engineering, Sungkyunkwan University, Suwon 16419, Republic of Korea

**Keywords:** silver nanoparticles, polyaspartamide, reactive oxygen species (ROS), oxidative stress

## Abstract

Recently, silver nanoparticles (AgNPs) have been extensively explored in a variety of biological applications, especially cancer treatment. AgNPs have been demonstrated to exhibit anti-tumor effects through cell apoptosis. This study intends to promote cell apoptosis further by increasing oxidative stress. AgNPs are encapsulated by biocompatible and biodegradable polyaspartamide (PA) (PA-AgNPs) that carries the anti-cancer drug Doxorubicin (Dox) to inhibit cancer cells primarily. PA-AgNPs have an average hydrodynamic diameter of 130 nm, allowing them to move flexibly within the body. PA-AgNPs show an excellent targeting capacity to cancer cells when they are conjugated to biotin. In addition, they release Dox efficiently by up to 88% in cancer environments. The DCFDA experiment demonstrates that the Dox-carried PA-AgNPs generate reactive oxidation species intensively beside 4T1 cells. The MTT experiment confirms that PA-AgNPs with Dox may strongly inhibit 4T1 cancer cells. Furthermore, the in vivo study confirms that PA-AgNPs with Dox successfully inhibit tumors, which are about four times smaller than the control group and have high biosafety that can be applied for chemotherapy.

## 1. Introduction

Cancer, according to the World Health Organization, is still a severe and challenging disease. Accurate cancer diagnosis and therapy are important to effectively preventing and eliminating cancer and maintaining a patient’s life. Surgery, chemotherapy, and radiation therapy were the most common treatments for cancer until recently. Chemotherapy is still the most effective treatment for many types of cancer [1,2]. However, owing to their non-specificity, they still have adverse side effects on patients after treatment [3]. New strategies for improving cancer therapies have been developed and are being researched based on the distinct features of cancer cells. Chemotherapy-associated oxidative stress, for example, relies on cancer cells being more vulnerable to reactive oxygen species (ROS) to eliminate cancer cells [4,5]. Due to their fast growth, cancer cells make more ROS than normal cells, so when ROS levels suddenly rise, cancer cells often get more damaged than normal cells [6]. Furthermore, combining these approaches with drug-delivery systems to deliver drugs to cancer sites and uptake into cancer cells has opened new avenues for cancer therapy. This delivery system requires materials of appropriate size and characteristics to move safely through the body. 

In the 21st century, nanomaterials have emerged as the material of the century [7]. Nanotechnology advancements have developed exciting chances to advance the efficacy of cancer treatments [8,9]. Drug-loaded nanoparticles with diameters ranging from 10 to 200 nm have many advantages over free drugs, including prolonged circulation, enhanced tumor target, increased cell accumulation, and the potential to combine diverse regimens, allowing for a combination treatment [10,11]. Silver nanoparticles with extraordinary features, such as good antibacterial activity, have been used in a variety of applications [12,13]. Because of their capacity to create ROS through a Fenton-like reaction, silver nanoparticles have recently been proposed as a possible agent for oxidative chemotherapy [14,15]. Silver nanoparticles could be used instead of or in combination with anti-cancer drugs to lower the dose while the drugs work better.

**Scheme 1:**
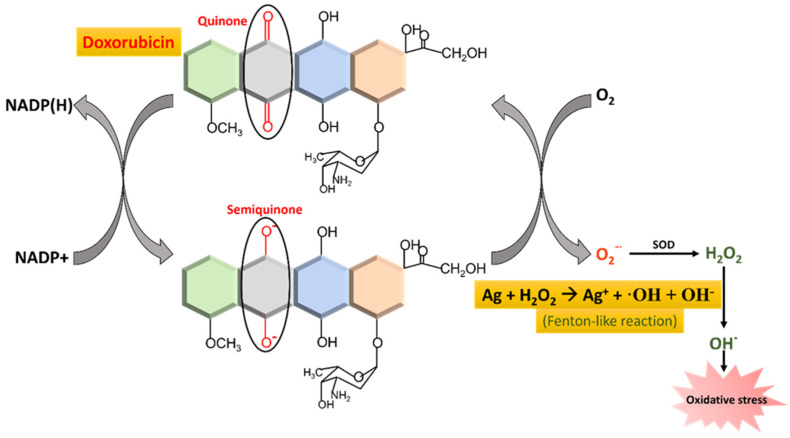
Oxidative stress generation mechanism.

Herein, the combination of AgNPs with Doxorubicin (anti-cancer drug) [16,17,18] to generate a potential material efficiently increases the amount of ROS in cancer cells, illustrated in Scheme 1. Dox and AgNPs are integrated using biocompatible and biodegradable polyaspartamide (PA). The PA carries and releases Dox into cancer cells through the pH-sensitive group hydrazine hydrate and encapsulates AgNPs via the hydrophobic interaction of the octadecylamine group (C18) on the PA and oleyamine on the AgNPs surface. In addition, biotin is conjugated with the PA to actively target cancer and uptake cancer cells, whereas polyethylene glycol (PEG) promotes hydrophilic and prolongs blood circulation time in the body.

## 2. Results and Discussion

### 2.1. Chemical Structure of PA

**Figure 1:**
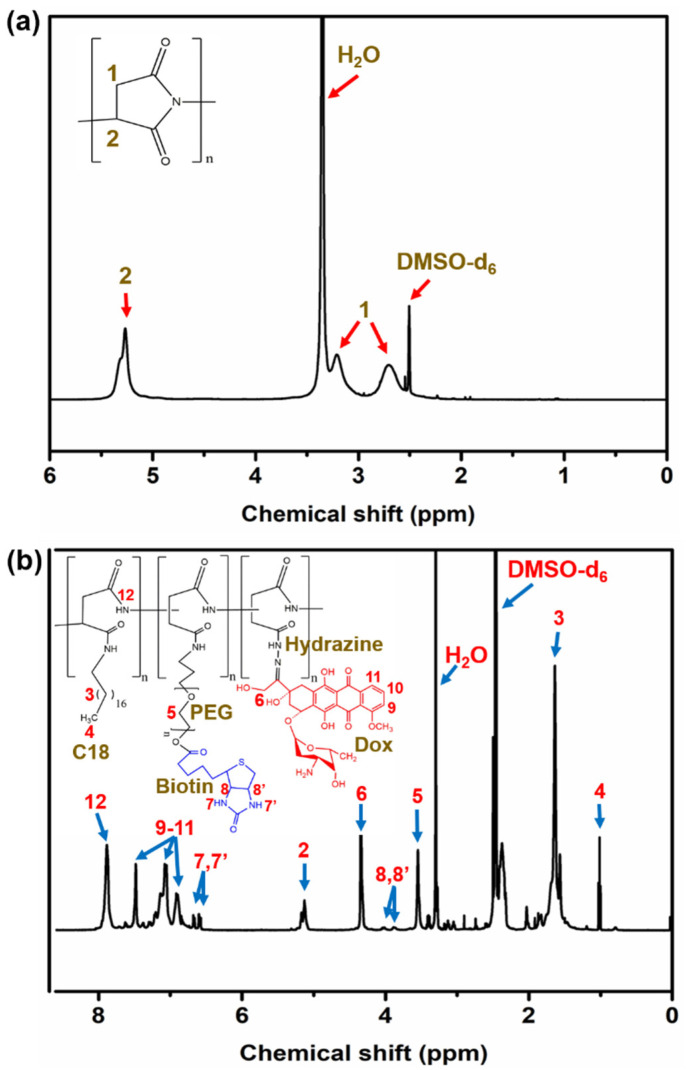
^1^H NMR spectra of (**a**) PSI and (**b**) PA.

Figure 1a shows the ^1^H NMR spectrum of PSI, a backbone of the PA. A signal at 5.1–5.3 ppm (number 2) indicated the methine protons, whereas two signals at 2.4–2.5 ppm and 3.2–3.5 ppm (number 1) identified the methylene groups [19,20]. The presence of these groups indicated full construction of the PSI backbone in the polymer chain. From *n* = 3.52 × η_red_^1.56^ (where decreased viscosity, η_red_ = 27), the degree of polymerization (*n*) was calculated to be 60,000 g mol^−1^ [21].

The PA was formed by grafting functional groups onto the PSI. Figure 1b shows the ^1^H NMR spectrum of the PA. Two proton signals at 1.63 (number 3) and 0.99 (number 4) demonstrated the presence of C18 [22]. Proton signals at 3.55 ppm (number 5) and 4.35 ppm (number 6) indicated the presence of PEG [23]. To target specific cancer cells, biotin was conjugated to PEG, which was detected by methane protons at 4.02 and 3.9 ppm (number 8, 8′) and urea protons at 6.67 and 6.6 ppm (number 7, 7′), respectively [24]. Dox was conjugated to PSI through the hydrazine hydrate group. The hydroxyls at 4.35 ppm (number 6) and aromatic rings at 7.45, 7.05, and 7.01 ppm (number 9–11) indicated the appearance of Dox [25]. A peak at 7.85 ppm (number 12) characterized the NH group of the PA (after ring-opening) and hydrazine hydrate [26]. Finally, the ^1^H NMR result showed that the PA grafted with a different functional group was successfully synthesized. The actual degree of substitution (DS) of C18, PEG, biotin, and Dox was roughly 9, 2, 20, and 50%, respectively, when the corresponding grafted levels were 10, 2.5, 100, and 100.

### 2.2. Characterization of PA-AgNPs

**Figure 2:**
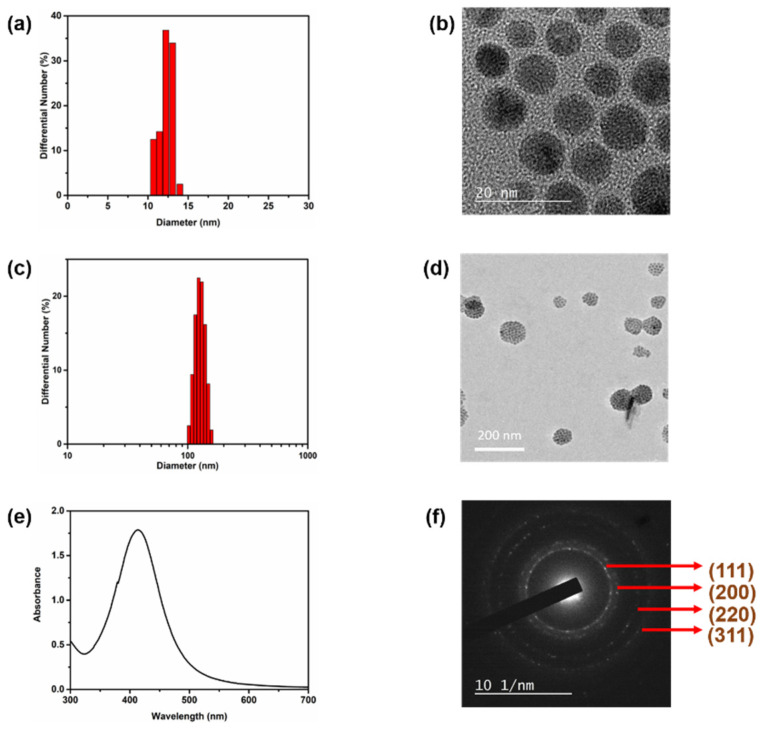
DLS size distribution of (**a**) AgNPs and (**c**) PA-AgNPs; TEM images of (**b**) AgNPs and (**d**) PA-AgNPs; (**e**) UV-Vis spectrum of AgNPs; and (**f**) electron diffraction (SAED) of PA-AgNPs.

From the DLS results (Figure 2a), as-synthesized AgNPs had average hydrodynamic diameters of 12 nm. The absorption peak at 420 nm in the UV-Vis spectrum (Figure 2e) confirmed the formation of AgNPs [27]. TEM images of the AgNPs (Figure 2b) showed uniform spheres with a diameter of 8 nm. AgNPs had average hydrodynamic diameters of 130 nm after they were encapsulated with the PA (Figure 2c). The TEM image of PA-AgNPs showed a grain diameter ranging from 50 to 100 nm (Figure 2d). PA-AgNPs with diameters less than 200 nm may be suitable for biomedical applications because they can move flexibly inside the body [28,29]. Electron diffraction patterns in Figure 2f also showed the structure of PA-AgNPs. The rings corresponded to (111), (200), (220), and (311), which specified for planes in the face-centered cubic (fcc) structure of Ag [30].

### 2.3. Cellular Uptake Ability

**Figure 3:**
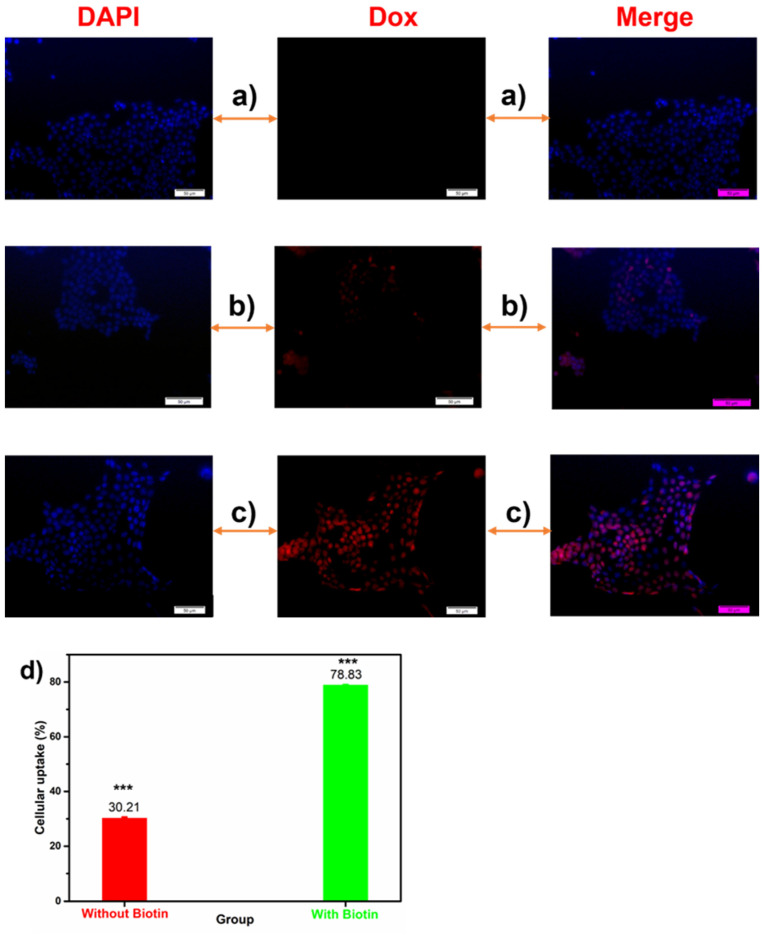
Cellular uptake and DAPI nuclei staining on 4T1 cells of (**a**) control, (**b**) PA-AgNPs without biotin, (**c**) PA-AgNPs with biotin; and (**d**) quantitative analysis of cellular uptake on 4T1 cells. (Data characterize the mean ± SD, *n* = 3, *** *p* < 0.001, Student’s *t*-test).

While all normal cells need enough vitamins to survive, cancer cells require a considerable number of vitamins to proliferate rapidly. As a result, the receptors on the cancer cell surface are overexpressed to capture as many vitamins as possible. Vitamin receptors may therefore be used to deliver tumor-targeting drugs. Biotin (vitamin H or B7) is a cellular growth promoter, and its amount in tumors is much higher than in normal tissues. Accordingly, biotin has gained popularity as a cancer cell-targeting agent [31]. In this study, the red signal fluorescent of Dox was used to evaluate the targeting ability of PA-AgNPs, while the blue signal fluorescent of DAPI was used to demonstrate the presence of cells. Figure 3a (control group) exhibited the blue signal associated with cell survival. Because the control group was not treated with PA-AgNPs, no red signal was seen. The group treated with PA-AgNPs without biotin showed just a few red signals (Figure 3b), but the group treated with biotin showed dense red signals (Figure 3c). Additionally, Figure 3d indicated that the quantity of PA-AgNPs was 2.6 times greater in the biotin group than in the non-biotin group. The results suggested that PA-AgNPs alone were ineffective in targeting cancer cells. Biotin, on the other hand, increased the uptake of nanoparticles into cancer cells.

### 2.4. In Vitro Drug Release

**Figure 4:**
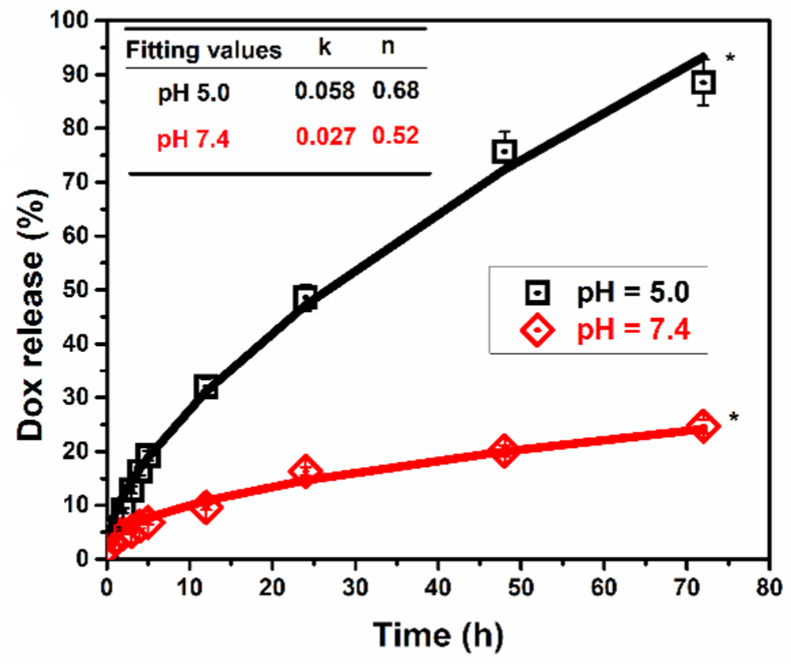
In vitro Dox release kinetics of PA-AgNPs at different pHs. (Data characterize the mean ± SD, *n* = 3, * *p* < 0.05, one-way ANOVA.) The solid curves are fitted to experiment data using Equation (1).

Figure 4 shows in vitro Dox release kinetics of PA-AgNPs at different pHs. The potential of PA-AgNPs to carry and release Dox was confirmed at pH 5.0 and 7.4, which characterized cancer cell lysosomes and physiological media, respectively [32]. About 24% of Dox was released at pH 7.4 and approximately 88% was released at pH 5.0, suggesting that hydrazone bonds were broken more effectively in acidic lysosomes than in physiological environments. The experimental data was fitted using Equation 1 to further define the drug-release mechanism:

M_t_/M_o_ = k t^n^ (1)

where M_t_ and M_o_ denote the released amount of drug (mass) during time t and the total quantity of drug (mass) loaded at the beginning of the release process; k is defined as a kinetic parameter that denotes the drug-release rate of a carrier in a certain environment, and *n* is the kinetics exponent that indicates the drug-releasing mechanism [33,34]. At pH 7.4, *n* was close to 0.5, implying that drug release was controlled by a Fickian diffusion mechanism. Dox was mostly released by physical diffusion along a concentration gradient. However, at pH = 5.0, the value of 0.5 ≤ *n* ≤ 1 indicated non-Fickian kinetics due to the hydrazone bond breaking under acidic environments [33,34]. These results showed that PA-AgNPs had great Dox-carrying and -releasing ability for cancer treatment.

### 2.5. Detection of Reactive Oxygen Species (ROS)

**Figure 5:**
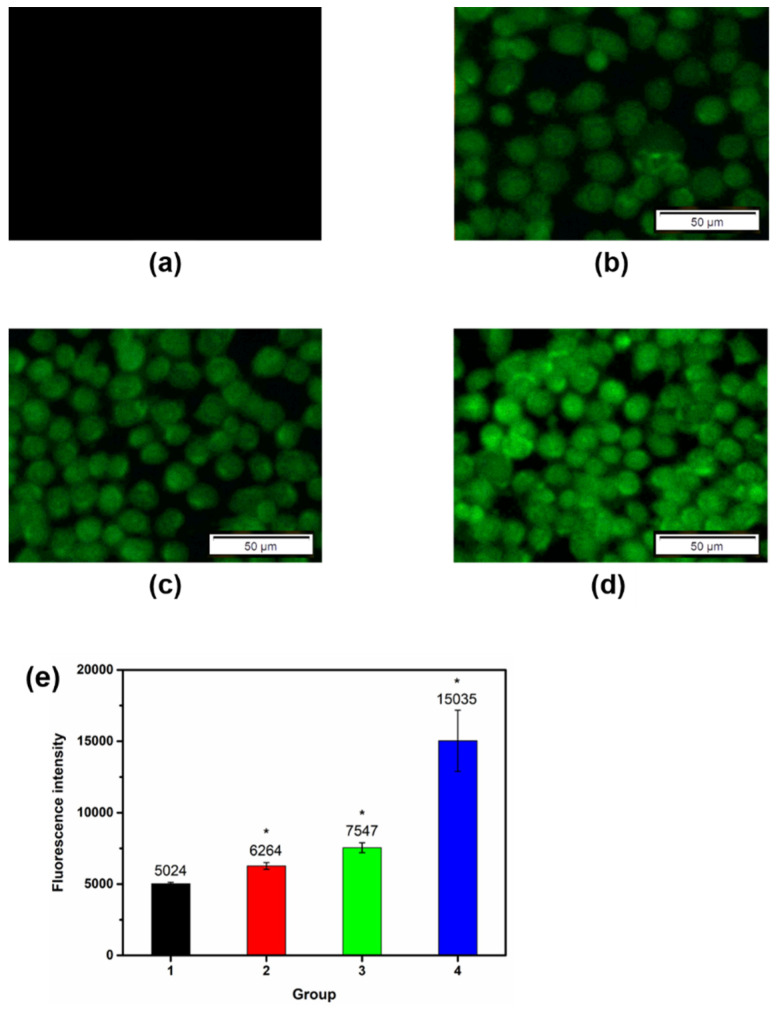
Detection of ROS using DCFDA assay on 4T1 cells for (**a**) control; (**b**) PA-AgNPs without Dox; (**c**) Dox-carried PA without AgNPs; (**d**) Dox-carried PA-AgNPs; and (**e**) fluorescence intensity of each group (Group 1: Control; Group 2: PA-AgNPs without Dox; Group 3: Dox-carried PA without AgNPs; Group 4: Dox-carried PA-AgNPs). (Data characterize the mean ± SD, *n* = 3, * *p* < 0.05, Student’s *t*-test).

When the amount of ROS is suddenly increased by external agents, oxidative stress occurs, leading to cancer cell death. The combination of AgNPs and Dox was expected to dramatically enhance ROS, as demonstrated by the DCFDA assay in this experiment (Figure 5). Cellular esterizes deacetylate DCFDA was used to produce non-fluorescent H_2_DCF after diffusing into cells. The amount of ROS may be estimated by the fluorescent intensity of DCF because ROS can oxidize H_2_DCF to form the highly green-fluorescent 2′,7′-dichlorofluorescein (DCF) [35]. The green signals of 4T1 cells were not significant (Figure 5a), indicating that the quantity of ROS produced in the cell was probably reduced by the antioxidant system. The green signals in Figure 5b were stronger than those in Figure 5a, indicating that the silver nanoparticles themselves may enhance ROS levels in cancer cells via the Fenton-like reaction [4,5]. As seen in Figure 5c, Dox-treated cells exhibited a significant increase in ROS when compared to the control group. Dox enhanced ROS through redox cycling via a quinone group in its chemical structure. Quinone was converted to semi-quinone free radicals by the respiratory chain complex I when Dox entered cancer cells. The semiquinone then gave O_2_ an electron, which caused the superoxide anion O^2¯˙^ to be formed. The antioxidant system then converted O^2¯˙^ to H_2_O_2_ [36]. Surprisingly, the combination of AgNPs and Dox generated a strong green signal, as seen in Figure 5d. Thus, a sudden increase in H_2_O_2_ caused by Dox, combined with the presence of AgNPs, triggered the Fenton-like reaction, resulting in a dramatic increase in ROS. The quantitative analysis, shown in Figure 5e, confirmed that Group 4 had the highest fluorescence intensity, which was 1.9, 2.4, and 2.9 times that of Groups 3, 2, and 1. These results demonstrated that Dox-carried PA-AgNPs could successfully induce ROS inside cancer cells, suggesting that they could be a promising anticancer agent.

### 2.6. In Vitro Cytotoxicity

**Figure 6:**
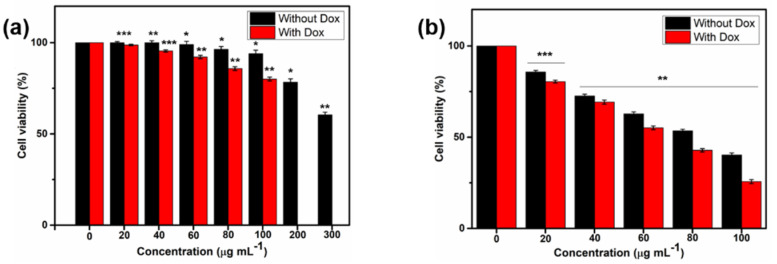
Cytotoxicity of PA-AgNPs with or without Dox (**a**) on 3T3 cells (**b**) on 4T1 cells. (Data characterize the mean ± SD, *n* = 6, * *p* < 0.05, ** *p* < 0.01, and *** *p* < 0.01, which were compared with the control group, Student’s *t*-test).

The efficacy of PA-AgNPs to suppress cancer cells was investigated using the MTT assay on 4T1 cancer cells. PA-AgNPs, however, must be safe for normal cells to be used in therapeutic treatment. As a result, the toxicity of PA-AgNPs was also investigated in the 3T3 normal cell line. As demonstrated in Figure 6a, PA-AgNPs (without/with Dox) concentrations ranging from 0 to 100 μg mL^−1^ did not seem to impair 3T3 cell viability due to poor uptake by normal cells. However, at doses more than 100 μg mL^−1^, it might be toxic to 3T3 cells (cell viability decreased to less than 80%). As a result, concentrations ≤ 100 μg mL^−1^ were applied for future research. Figure 6b shows that silver nanoparticles or Dox may dramatically suppress cancer cells. Cell viability was lowered more than in the case of AgNPs alone when combined with Dox at the same concentration. This was because the mechanism of ROS generation was explained in Section 2.5. These results suggested that Dox-carried PA-AgNPs were both safer and more effective for cancer prevention.

### 2.7. Cell Apoptosis Assay

**Figure 7:**
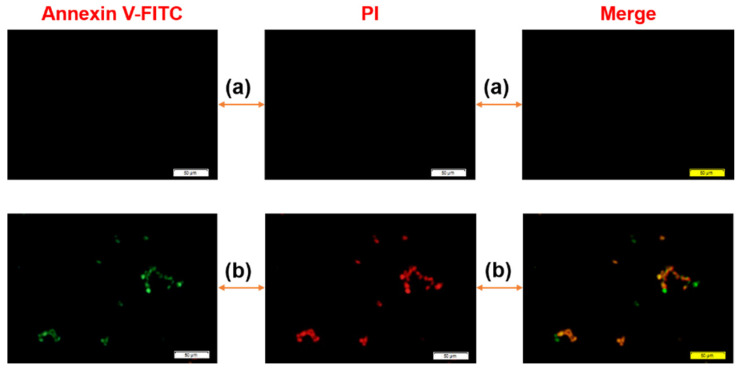
Fluorescent microscopic images of Annexin V-FITC/PI on 4T1 cells with (**a**) control and (**b**) PA-AgNPs.

When cells are exposed to oxidative stress, they could undergo apoptosis. The co-staining annexin V-FITC kit with PI was used to identify the mechanism of cancer cell death in Section 2.6. The cell membrane changed during early apoptosis, exposing phosphatidylserine (PS) to the surface. Annexin V-FITC can bind PS to detect early apoptosis (green signals). When a cell is in late apoptosis or necroptosis, the cell membrane is ruptured, allowing the PI to penetrate and bind to DNA, giving it a red signal [37]. Thus, healthy cells have both annexin V and PI signals that are negative, whereas cells in early apoptosis have annexin V-positive but no PI signals, and cells in late apoptosis have both annexin V and PI signals that are positive [38]. The healthy cells in the control group showed no fluorescence signal (Figure 7a), whereas the cells treated with PA-AgNPs showed early apoptosis (green signals–Figure 7b). Furthermore, many cells showed both signals, suggesting that they were in late apoptosis (Figure 7b). According to the results, PA-AgNPs possessed cancer-inhibitory potential through the oxidative stress pathway.

### 2.8. In Vivo Therapy Studies

**Figure 8:**
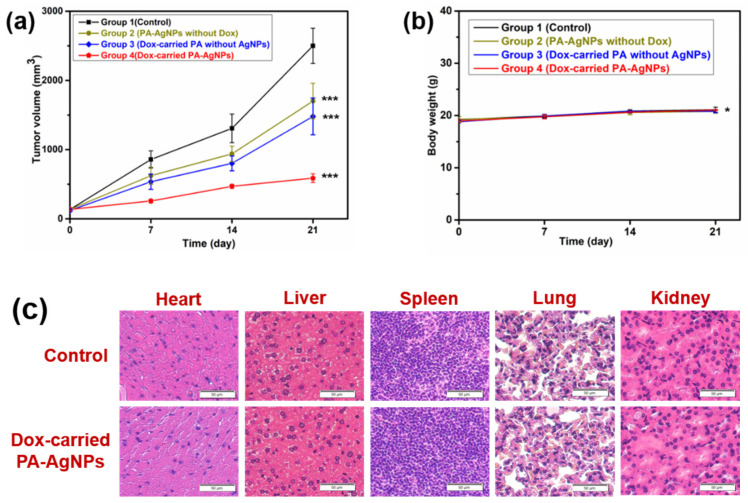
(**a**) In vivo antitumor efficacy of PA-AgNPs; (**b**) body weight change of groups as a function of time (Data characterize the mean ± SD, *n* = 3, * *p* < 0.05 and *** *p* < 0.01 compared with the control group, Student’s *t*-test.); and (**c**) H&E staining of major organs.

The tumor-suppressing ability of PA-AgNPs has been demonstrated in vivo (Figure 8a). 4T1 tumor-bearing mice with a volume of 100–150 mm^3^ were injected with an IV with different materials. After the 21-day treatment, the tumors of the mice in the control group increased the most, reaching nearly 2500 mm^3^. The tumor growth of mice treated with only AgNPs or Dox particles was weaker than that of the control group. Especially, in the group treated with Dox-carried PA-AgNPs, the tumor volume increased very little and was four times smaller than that of the control group. These results suggested that PA-AgNPs played a significant role in anticancer drug-delivery systems, showing an excellent performance in vitro and in vivo and suggesting therapeutic effectiveness.

### 2.9. Biosafety In Vivo Studies

In order to have a great clinical application, the material should have biosafety properties in addition to therapeutic effectiveness. As a result, PA-AgNPs were evaluated for toxicity in vivo by measuring body mass changes in groups of treated mice as well as histological changes in the main organs of treated mice. Figure 8b shows the increase in body weight over time in all groups of mice, suggesting that PA-AgNPs had insignificant side effects [39]. Furthermore, there were no significant differences in histological abnormalities in organs such as the liver, lung, heart, kidney, and spleen between the PA-AgNPs group and the control group (Figure 8c). These results indicated that PA-AgNPs were biosafe and have the potential to be used in chemotherapy [40].

## 3. Materials and Methods

### 3.1. Materials

The materials for the experiments are listed in Table 1. These materials were obtained from a variety of commercial sources, including Sigma-Aldrich (Milwaukee, WI, USA). The following critical processes were included in the experimental procedure: the synthesis of silver nanoparticles (AgNPs), the synthesis of PA, and the encapsulation of AgNPs with PA.

### 3.2. Synthesis of Silver Nanoparticles

Silver acetate (50 mg) and oleyamine (2 g) were dissolved in toluene (50 mL) and stirred for 24 h. The color of the mixture solution slowly turned dark brown. After the reaction, the solution was concentrated to about 15 mL. The AgNPs were collected by adding 200 mL of ethanol, centrifuged at 10,000 rpm, and washed three times with ethanol.

### 3.3. PA Synthesis

Polysuccinimide was first synthesized to play as the polyaspartamide backbone. L-aspartic acid (25 g), sulfolane (30 g), mesitylene (70 g), and a phosphoric acid catalyst (0.643 mL) were stirred together for 8 h at 170 °C in nitrogen gas. A Dean-Stark trap was used to obtain the reaction by-product (water). After the reaction, the product was filtered, washed many times with DIW and methanol, and dried at 70 °C in a vacuum oven. The functional groups were then grafted onto PSI. Octadecylamine (0.2776 g), o-(2-aminoethyl) polyethylene glycol (PEG), hydrazine hydrate (Hyd), and PSI (1 g) were dissolved in 30 mL DMF and stirred in nitrogen gas for 48 h. To obtain the product, the post-reaction mixture was dialyzed and freeze-dried.

Biotin was conjugated with PEG by stirring a mixture of Hyd/PEG/C18-PSI (0.5 g), biotin (0.0005 g), and DMF (10 mL) at 0 °C (5 min) and room temperature (RT-24 h). *N*,*N*′-dicyclohexylcarbodiimide (DCC) (0.0208 g) and 4-(dimethylamino) pyridine (DMAP) (0.0011 g) were used as catalysts. After the reaction, the solvent was removed using a dialysis membrane and the product was obtained after freeze-drying. Dox was conjugated to hydrazine hydrate. Dox (0.0004 g), triethylamine (2.16 μL), and dimethyl sulfoxide (20 mL-DMSO) were stirred in the dark at RT. After that, the Hyd/PEG-Biotin/C18-PSI (0.01 g) was added and stirred for 48 h. Dialysis and freeze-drying were conducted to collect the final product.

### 3.4. Encapsulation of AgNPs with PA

AgNPs were dispersed in tetrahydrofuran (1 mg mL^−1^), while the PA was dissolved in DMF (10 mg mL^−1^). Distilled water (DIW, 30 mL) was added to the AgNPs solution and sonicated for 20 min. After adding the PA solution to the AgNPs solution, sonication was continued for 10 min. PA-AgNPs were collected by washing the solution many times with DIW, centrifuging at 10,000 rpm, and drying.

### 3.5. Physicochemical and Structural Characterizations

The morphology and structure of PA-AgNPs were studied using high-resolution transmission electron microscopy (HR-TEM; JEM-2100 F, JEOL, Akishima, Japan). The hydrodynamic diameter of PA-AgNPs was confirmed by dynamic light scattering (DLS; ELS-Z model, Otsuka, Japan). ^1^H NMR spectroscopy (Unity Inova 500 MHz, Varian, Palo Alto, CA, USA) was employed to check the molecular structure of PA.

### 3.6. In Vitro Drug Release

An in vitro drug release experiment was conducted using a dialysis method. The dialysis membrane was initially soaked in DIW for 15–30 min (molecular weight cut-off of 6–9 kDa). The sample (10 mg mL^−1^) was dissolved in PBS (pH 7.4) before being put in a dialysis tube. After that, the dialysis tube was put in a vial containing 40 mL of PBS and slightly stirred at 37 °C. After 1, 2, 3, 4, 5, 12, 24, 48, and 72 h, 1 mL of the PBS was taken from the vial and 1 mL of new PBS was added to the vial. To determine the concentration of Dox released, the absorption intensity of the taken PBS was measured at 482 nm using UV-visible spectrophotometry. The pH 5.0 sample was similarly handled to the pH 7.4 sample. The absorption intensities of the free Dox solution at different concentrations were used to determine the standard calibration curve for detecting the concentration of Dox release. 

### 3.7. Cell Viability

The cell viability and cytotoxicity of PA-AgNPs with and without Dox were assessed using the MTT (3-(4,5-dimethylthiazol-2-yl)-2,5-diphenyltetrazolium bromide) assay (Sigma-Aldrich, Milwaukee, WI, USA). 1 × 10^4^ of 4T1 breast cancer cells were grown in 96-well microplates for 24 h in the incubation machine. PA-AgNPs with or without Dox at concentrations ranging from 20 to 300 μg mL^−1^ were applied to the microplates and continued to incubate for 24 h. After that, the wells were poured with 20 μL of MTT solution (5 mg mL^−1^) and grown for 4 h. After removing the medium, each well received 200 μL of DMSO and was incubated for 30 min. A multiskan microplate (Thermal Fisher Scientific, Finland) was used to check the cells at 490 nm. For comparison, the same processes were performed on 3T3 normal cells.

### 3.8. Cellular Uptake

The cellular uptake of PA-AgNPs was determined using the Dox fluorescence signal. 3 × 10^4^ 4T1 cells were grown with a culture medium in a 6-well plate and incubated for 24 h. PA-AgNPs were given to each well at a concentration of 100 µg mL^−1^. After 24 h of incubation in the dark, the culture medium was taken out and the cells were washed twice in PBS. Triton X-100 permeated the cells, which were then fixed with 4% paraformaldehyde. After that, the cells were stained for 5 min with 20 nM 4′,6-diamidino-2-phenylindole (DAPI) (Sigma-Aldrich, Milwaukee, WI, USA) before being examined under fluorescence microscopy. The Cytation 5 plate reader was used for quantitative analysis (Biotek Instruments, Inc., Winooski, VT, USA).

### 3.9. Detection of Reactive Oxygen Species (ROS)

The DCFDA (2′,7′-dichlorofluorescein diacetate) assay (Sigma-Aldrich, Milwaukee, WI, USA) was used to assess intracellular ROS generation. 3 × 10^4^ 4T1 cells were grown in 6-well plates for 24 h at 37 °C. Following that, the samples (100 μg mL^−1^) were added, and the grow time was increased. After 24 h of incubation, the cells were washed twice with PBS before being exposed to 20 μM DCFDA for 45 min in the dark. The cells were rinsed twice with PBS before using fluorescence microscopy. A Cytation 5 plate reader was used for quantitative analysis.

### 3.10. Cell Apoptosis Assay

To evaluate cell apoptosis, an annexin V-FITC apoptosis detection kit with propidium iodide (PI) was employed (Thermo fisher scientific, Australia). 2 × 10^5^ 4T1 cells were grown in 6-well plates at 37 °C for 24 h. After that, the PA-AgNPs (80 μg mL^−1^) were added to the plates and incubated. The cells were rinsed twice with PBS after 24 h of incubation and collected using 200 μL of Trypsin-EDTA solution (0.25%). After that, the cells were suspended in 190 μL of 1X binding buffer. 5 μL of annexin V-FITC was added and maintained at room temperature in the dark for 5 min. 5 μL of PI was then added and continued to be maintained for 5 min. The cells were collected by centrifuging and suspended in 200 μL of 1X buffer for fluorescence microscopy.

### 3.11. In Vivo Experiments

Sungkyunkwan University’s Institutional Animal Care and Use Committee approved the protocol for all animal studies (IACUC). Hanlim Experimental Animal Center (Seoul, South Korea) supplied six-week-old female BALB/c mice. 5 × 10^5^ 4T1 cells were subcutaneously injected into the backs of mice to form tumors. When the tumor volume reached 100–150 mm^3^, the mice were divided into four groups of three animals each. For three weeks, four groups received intravenous injections of various solutions once a week: Group 1 used a PBS solution as a control, Group 2 used PA-AgNPs without Dox, Group 3 used a Dox-carried PA without AgNPs, and Group 4 used Dox-carried PA-AgNPs. The dose of the injection was 0.1 mg kg^−1^ of body weight. Every two days, the tumor size was measured using a digital caliper. V = length × (width)^2^ × 0.5 [41] was used to compute tumor volumes (V). Body weight was measured at the same time.

Following the completion of the treatment period (3 weeks), the organs of the heart, lung, spleen, liver, and kidney were sacrificed and preserved in 4% paraformaldehyde for histological analysis. Before sectioning the organs into 5 μm-slides, they were paraffinized. Hematoxylin and eosin (H&E) staining were used on the slides, which were then analyzed under a microscope.

### 3.12. Statistical Analysis

Origin 9.1, Graphpad Prism 9.0, and Image J software were used for statistical analysis. The *p* value was statistically significant with * *p* < 0.05, ** *p* < 0.01, and *** *p* < 0.001 using Student’s *t*-test and one-way analysis of variance (ANOVA).

## 4. Conclusions

AgNPs and Dox were integrated in a biocompatible and biodegradable polyaspartamide to form a potential material that could suppress cancer predominantly via the oxidative stress pathway. The diameter of the synthesized AgNPs was around 8 nm, whereas the average hydrodynamic diameter of the PA-AgNPs was 130 nm. Biotin effectively increased cancer cell uptake, resulting in a significant amount of ROS being created in 4T1 cancer cells, as revealed by the DCFDA assay. As a result, as indicated by the MTT and apoptosis assays, PA-AgNPs could effectively inhibit cancer cells by inducing apoptosis in cancer cells. An in vivo experiment with a tumor volume about four times less than that of the control group confirmed tumor-suppressing effectiveness of PA-AgNPs. Furthermore, in vivo biosafety experiments revealed that PA-AgNPs were highly biosafe. As a result, PA-AgNPs showed substantial potential for chemotherapy.

## Figures and Tables

**Table 1:** Summary of all materials used.

Synthesis Process	Material		Function	Status
	Name	Chemical Formula/Dilution		
PSI	*N*,*N*’-dimethylformamide	DMF, 99.8%	Solvent	Solution
	L-Aspartic acid	98%	Substrate	Powder
	Phosphoric acid	85%	Catalyst	Solution
	Mesitylene	98%	Solvent	Solution
	Sulfolane	99%	Solvent	Solution
PA	Octadecylamine	C18, 99%	Substrate	Powder
	*O*-(2-aminoethyl)polyethylene glycol	PEG, Mp 5000	Substrate	Powder
	Biotin	≥99%	Substrate	Powder
	Hydrazine hydrate	50–60%	Substrate	Solution
	Doxorubicin hydrochloride	98–102%	Substrate	Powder
	*N,N’-*dicyclohexincarbodiimide	99%	Catalyst	Powder
	4-(Dimethylamino)pyridine	≥99%	Catalyst	Powder
	Triethylamine	≥99.5%	Catalyst	Solution
	Dimethyl sulfoxide	DMSO, ≥99%	Solvent	Solution
AgNPs and Encapsulation	Toluene	≥99.8%	Solvent	Solution
	Distilled Water	DIW	Solvent	Solution
	Oleyamine	99%	Surfactant	Solution
	Tetrahydrofuran	99%	Solvent	Solution
	Silver acetate	99%	Substrate	Powder

## Data Availability

Data is contained within the article.
